# Pneumococcal conjugate vaccine primes mucosal immune responses to pneumococcal polysaccharide vaccine booster in Papua New Guinean children

**DOI:** 10.1016/j.vaccine.2020.10.042

**Published:** 2020-11-25

**Authors:** Tilda Orami, Rebecca Ford, Lea-Ann Kirkham, Ruth Thornton, Karli Corscadden, Peter C. Richmond, William S. Pomat, Anita H.J. van den Biggelaar, Deborah Lehmann, for the Neonatal Pneumococcal Conjugate Vaccine Trial team

**Affiliations:** a Papua New Guinea Institute of Medical Research, Goroka, Eastern Highlands Province, Papua New Guinea; b Wesfarmers Centre of Vaccines and Infectious Diseases, Telethon Kids Institute, Perth, Western Australia, Australia; c Centre for Child Health Research, The University of Western Australia, Perth, Western Australia, Australia; d School of Biomedical Sciences, Faculty of Health and Medical Sciences, The University of Western Australia, Perth, Western Australia, Australia; e Division of Pediatrics, School of Medicine, The University of Western Australia, Perth, Western Australia, Australia

**Keywords:** Pneumonia, Pneumococci, IgA, IgG, Salivary antibodies, Papua New Guinea, Children, Pneumococcal conjugate vaccine, Pneumococcal polysaccharide vaccine, Immune memory, Mucosal immunity

## Abstract

**Introduction:**

Invasive pneumococcal disease remains a major cause of hospitalization and death in Papua New Guinean (PNG) children. We assessed mucosal IgA and IgG responses in PNG infants vaccinated with pneumococcal conjugate vaccine (PCV) followed by a pneumococcal polysaccharide vaccine (PPV) booster.

**Methods:**

Infants received 7-valent PCV (7vPCV) in a 0–1–2 (neonatal) or 1–2-3-month (infant) schedule, or no 7vPCV (control). At age 9 months all children received 23-valent PPV (23vPPV). IgA and IgG to 7vPCV and non-7vPCV (1, 5, 7F, 19A) serotypes were measured in saliva collected at ages 1, 2, 3, 4, 9, 10 and 18 months (131 children, 917 samples). Correlations were studied between salivary and serum IgG at 4, 10 and 18 months.

**Results:**

Salivary IgA and IgG responses overall declined in the first 9 months. Compared to non-7vPCV recipients, salivary IgA remained higher in 7vPCV recipients for serotypes 4 at 3 months, 6B at 3 months (neonatal), and 14 at 3 (neonatal), 4 and 9 months (infant); and for salivary IgG for serotypes 4 at 3, 4 and 9 months, 6B at 9 months, 14 at 4 (neonatal) and 9 months, 18C at 3, 4, and 9 (infant) months, and 23F at 4 months. Following 23vPPV, salivary 7vPCV-specific IgA and IgG increased in 7vPCV-vaccinated children but not in controls; and salivary IgA against non-PCV serotypes 5 and 7F increased in 7vPCV recipients and non-recipients. Salivary and serum IgG against 7vPCV-serotypes correlated in 7vPCV-vaccinated children at 4 and 10 months of age.

**Conclusions:**

PCV may protect high-risk children against pneumococcal colonization and mucosal disease by inducing mucosal antibody responses and priming for mucosal immune memory that results in mucosal immune responses after booster PPV. Saliva can be a convenient alternative sample to serum to study PCV-induced systemic IgG responses.

## 1 Introduction

*Streptococcus pneumoniae* (pneumococcus) is the leading cause of pneumonia deaths in children under 5 years of age [1]. The pneumococcus is also a leading cause of meningitis, bacteraemia and otitis media [2]. Young children in the highlands of Papua New Guinea (PNG), where this study was performed, experience one of the highest rates of pneumococcal disease in the world, with approximately 5 out of 100 children experiencing invasive pneumococcal disease (IPD) in the first year of life [3].

*S. pneumoniae* is a common colonizer of the nasopharynx of children [4]. Pneumococcal colonization is a major risk factor for mucosal and invasive pneumococcal disease [5]. In the highlands of PNG, pneumococcal colonization starts within a few weeks of birth, with half of children being colonized before 3 weeks of age [6]. In addition, the range of colonizing pneumococcal serotypes is broad and more diverse than in low-endemicity settings and children can carry multiple serotypes at the same time [7], [8].

Nasopharyngeal colonization also drives immune development. The mucosal immune system rapidly develops shortly after birth in response to bacterial colonization [9], [10]. Immunoglobulin A (IgA) is the major class of antibodies found in mucosal secretions. The development of specific secretory (S)IgA antibodies in infants depends on the degree of natural exposure or vaccination. In a low-endemicity setting, infants as young as 6 months old were shown to have elevated serotype-specific salivary IgA antibodies if they had previously been colonized with pneumococci of the same serotype [11]. This early mucosal IgA response may protect children against subsequent carriage and potential disease of the same pneumococcal serotype [12], [13], [14]. It is not known whether children in high-endemicity settings, where colonization occurs at a younger age, also produce potentially protective mucosal antibody responses.

Pneumococcal conjugate vaccines (PCVs) induce systemic immune responses that are highly effective in preventing IPD due to vaccine serotypes in children in both low- and high-risk settings [15], [16]. In low-endemicity settings PCVs also reduce vaccine-serotype-specific carriage and mucosal infections [17], [18] and specific IgA and IgG antibodies can be detected in saliva of children who have completed their primary PCV immunizations, although levels vary between vaccine serotypes and between children [19], [20], [21]. Subsequent booster vaccination with pneumococcal polysaccharide vaccine (PPV) or PCV was shown to increase salivary IgA responses for PCV serotypes, which has led to the suggestion that primary PCV vaccination induces mucosal immune memory [19], [20], [21].

In contrast to low-endemicity settings, PCVs offer no or limited protection against pneumococcal colonization in high-endemicity settings [3], [22], [23], which raises questions about the induction of mucosal immunity by PCV under these conditions. Apart from a small study that measured salivary IgA responses in ten high-risk Aboriginal Australian children primed with PCV followed by a PPV booster [24], there are to our knowledge no studies on the development of salivary antibodies following primary and booster vaccination with pneumococcal vaccines in high-risk children. Whether PCV can induce mucosal immunity or prime for mucosal immune memory in children in high-endemicity settings is important to our understanding of the epidemiology of pneumococcal disease after implementation of PCV and for optimizing vaccination schedules. This includes understanding mucosal immune responses to a PCV-prime and PPV-boost schedule as a potential strategy to protect high-risk children against replacement carriage and disease.

The objectives of this study were to assess the induction, kinetics and persistence of mucosal IgA and IgG responses in children in the highlands of PNG in the first months of life in the absence or presence of primary vaccination with PCV; after vaccination with PPV in later infancy; and to study correlations between mucosal and systemic IgG responses after PCV primary immunization and PPV vaccination. These analyses were conducted in saliva and serum samples collected from children participating in a randomized placebo-controlled trial of accelerated 7-valent PCV (7vPCV) vaccination (Neonatal Pneumococcal Conjugate Vaccine Trial) conducted in the highlands of PNG between 2005 and 2009 [25]. We previously reported results for serum serotype-specific IgG responses following 7vPCV-priming and 23-valent PPV (23vPPV) booster vaccination, showing that PCV is immunogenic and induces systemic memory responses, but has limited impact on pneumococcal carriage in these high-risk children [3], [4].

## 2 Methods

### 2.1 Study design, participants and vaccines

The Neonatal Pneumococcal Conjugate Vaccine (NPCV) trial was an open-label randomized placebo-controlled trial of 7vPCV (Prevnar™, Pfizer) conducted in the Asaro Valley, Eastern Highlands Province of PNG. The overall study design, process of assent and consent, enrolment, immunization and follow-up have been reported in detail earlier [3], [25]. Briefly, 312 eligible newborns were enrolled between May 2005 and September 2007. Inclusion criteria were birth weight > 2000 g; no acute neonatal infection; no severe congenital abnormality; and born in Goroka General Hospital (GGH) or brought to GGH within 24 h of birth. Children of mothers known to be HIV-positive were excluded.

Infants were randomized 1:1:1 to receive 3 doses of 7vPCV by intramuscular injection in the left anterolateral thigh at 0, 1, and 2 months of age (neonatal group, n = 101) or at 1, 2, and 3 months of age (infant group, n = 105), or did not receive 7vPCV (control group, n = 106) [25], [26]. Each 0.5 mL dose of 7vPCV (Prevnar^TM^, Pfizer, batch numbers 15,422 and 21933) contained 2 μg of each polysaccharide for serotypes 4, 9V, 14, 18C, 19F, 23F and 4 μg for 6B coupled to CRM_197_ carrier protein, and 0.125 mg aluminium adjuvant. PCV was neither available nor part of the national immunization schedule in PNG at the time of this trial. Since PPV contains more pneumococcal serotypes than PCV, it could provide additional protection to high-risk children who suffer from disease due to a broad range of serotypes. Earlier studies in PNG demonstrated reduced pneumonia mortality in children given PPV between 6 months and 5 years of age [27]. Hence the safety and immunogenicity of PPV following PCV primary immunization or given on its own (control group) was investigated. All study children received one dose of 23vPPV (Pneumovax 23™, Merck & Co, batch number G3836) (containing 25 μg of each purified capsular polysaccharide for serotypes 1, 2, 3, 4, 5, 6B, 7F, 8, 9N, 9V, 10A, 11A, 12F, 14, 15B, 17F, 18C, 19A, 19F, 20, 22F, 23F and 33F) at 9 months of age by intramuscular injection in the left anterolateral thigh. Study children also received all childhood vaccines that were part of the PNG national immunization schedule at the time of the study (Bacillus Calmette-Guérin vaccine at birth; oral polio vaccine at birth, 1, 2 and 3 months; Hepatitis B vaccine at birth, 1 and 3 months; a combined diphtheria, tetanus, whole cell pertussis, *Haemophilus influenzae* type b vaccine at ages 1, 2 and 3 months and measles vaccination at 6 and 9 months) [28].

Study children were followed until 18 months of age. Of the 312 newborns, 240 completed the 18-month study according to protocol (78 in the neonatal group; 87 in the infant group; and 75 in the control group) [3].

### 2.2 Ethical considerations

Ethical approval was received from the Medical Research Advisory Committee of PNG (MRAC#: 03/02) and the Princess Margaret Hospital Ethics Committee in Perth, Australia (#1038EP). This trial is registered at Clinical Trials.gov under registration number NCT00219401 (http://clinicaltrials.gov/ct2/show/NCT00219401).

### 2.3 Specimen collection

Saliva samples were collected from infants at 1, 2, 3, 4, 9, 10 and 18 months of age. Mothers were asked to refrain from breastfeeding for 30–60 min prior to saliva sample collection to minimize contamination of saliva samples with maternal breastmilk IgA. In line with earlier validated protocols [24], saliva samples were collected by placing surgical eye spears (up to 6 per child per study visit) (Defries Industries, Victoria, Australia) in the cheeks and under the tongue for 3–5 min until completely soaked with saliva. Spears were transported in 15 mL falcon tubes to the PNG Institute for Medical Research laboratory on ice, where they were centrifuged to separate saliva. Saliva samples were then stored at –70 °C.

Samples to be analysed for serotype-specific IgA and IgG were selected based on a study participant having a complete set of samples. Samples were shipped in liquid nitrogen to Perth, Australia, for serotype-specific immunoassays.

### 2.4 Multiplex immunoassay for salivary serotype-specific IgG and IgA

Salivary IgG and IgA antibody levels against 7vPCV pneumococcal serotypes (4, 6B, 9V, 14, 18C, 19F, and 23F) and non-7vPCV serotypes 1, 5, 7F and 19A were measured using a multiplex fluorescent bead-based assay as described previously [29], [30], [31]. Fluorescence was measured using the Bioplex 200 System (Bio-Rad) and Bio-Rad Manager Software version 5.0 (Bio-Rad, CA, USA). Samples above the upper limit of quantification (LOQ) were re-tested for the given serotype(s) at higher dilutions. Samples below the lower LOQ were assigned half the detection limit concentration for the given serotype. (LOQ IgG range from 0.005 ng/mL to 0.045 ng/mL, IgA 0.035 ng/mL to 0.115 ng/mL). Assay reproducibility was confirmed by calculating % coefficient of variation (CV) of quality controls (QCs) over all assays.

### 2.5 Total protein assay

Total protein levels in saliva samples were measured as previously described [30] to correct for differences in salivary stimulation (which may occur for example as a result of food or drink intake) and time of collection. Salivary antibody was standardized as µg per mg of total protein in the saliva.

### 2.6 Lactose assay for the detection of breast milk in saliva

Potential contamination of saliva samples by breast milk antibodies (IgA) was tested by measuring lactose levels. Lactase reagent (0.1 mM magnesium chloride and 0.1 M phosphate buffer, pH 7.2) with and without β-galactosidase (8 units/mL), was prepared and 50 μL added per well such that each sample and standard was tested with and without β-galactosidase to determine background glucose levels. Lactose standards were prepared from α-lactose powder (Sigma Aldrich, St Louis) dissolved in sterile water (Baxter) such that final concentrations of 400 mM, 320 mM, 240 mM, 160 mM, 80 mM, 40 mM and 20 mM were achieved in the microtitre plate wells. 5 μL of each standard and saliva sample were added to a well with and without β-galactosidase. After incubation for 1 h at 37 °C, 50 μL of glucose reagent made from glucose oxidase (Roche Diagnostics, USA) and peroxidase type II from horseradish (Sigma-Aldrich, St Louis) was reduced in phosphate buffer solution. Saliva samples were added to the wells containing lactose with β-galactosidase (Roche, Australia) or without β-galactosidase and the optical densities were read at 405 nm and 650 nm as a reference. Samples with > 200 mM of lactose were considered positive for breast milk contamination and were excluded from IgA antibody data analyses.

### 2.7 Systemic serotype-specific IgG concentrations

Venous or capillary blood samples were collected from study children at 2, 3, 4, 9, 10 and 18 months of age. Serum serotype-specific IgG concentrations to 7vPCV serotypes and to non-7vPCV serotypes 2, 5 and 7F were measured using a WHO standardized pneumococcal enzyme-linked immunosorbent assay (ELISA) [32], and reported previously [3]. For this study, serum pneumococcal serotype-specific IgG responses measured at 4 months of age (1 and 2 months after completion of infant and neonatal PCV schedules, respectively), 10 months of age (1 month after 23vPPV), and 18 months of age (latest time point of follow-up for persistence of antibody) were used for correlation studies with corresponding salivary IgG responses.

### 2.8 Statistical analysis

Statistical analysis was performed using IBM SPSS Statistics 25. Antibody data were log-transformed. Geometric mean concentrations (GMCs) and proportions of children with detectable serotype-specific IgA and IgG antibodies, including 95% confidence intervals (95% CI), were calculated for the groups at different time points; differences between 7vPCV-vaccinated groups and the control group were considered significant when 95% confidence intervals did not overlap. Fold-changes in antibody responses after 23vPPV vaccination were calculated for the different groups by taking the geometric mean of individual ratios of 10-month versus 9-month antibodies (geometric mean ratio, GMR), and considered to be significant increases (GMR > 1) or decreases (GMR < 1) when 95% CIs did not include 1. For other timepoints, changes in antibody levels compared to baseline (at 1 month of age) were tested using the Wilcoxon signed-rank test. Correlations between serotype-specific IgG concentrations in saliva and serum samples collected at the same time points were assessed using the Spearman rank correlation test.

## 3 Results

### 3.1 Study population characteristics

Complete sets of 7 saliva samples were available for 131 of 312 children participating in the vaccination trial, of whom 42 were vaccinated with 7vPCV according to the 0–1–2-month schedule (neonatal 7vPCV), 51 according to the 1–2-3-month schedule (infant 7vPCV) and 38 who had not received 7vPCV (control group). Baseline characteristics of children (and their mothers) included in this analysis were comparable to the main study (Table 1).

**Table 1:** Characteristics of the children and their mothers included in this subgroup analysis and the main trial.

	Subpopulation current analysis			Main trial population		
	7vPCV groups			7vPCV groups		
	Neonatal (N = 42)	Infant (N = 51)	Control (N = 38)	Neonatal (N = 101)	Infant (N = 105)	Control (N = 106)
Age mother, years	25.9 (5.6)	25.1 (4.7)	25.4 (6.7)	25.7 (5.6)	25.3 (5.6)	25.4 (5.6)
Gender, male (%)	41%	51%	63%	43%	57%	61%
Gestational age, weeks	39.3 (1.3)	39.6 (1.2)	39.7 (1.1)	39.4 (1.2)	39.5 (1.3)	39.6 (1.0)
Birth weight, g	3184 (4 8 8)	3330 (4 2 4)	3384 (4 7 5)	3245 (4 4 0)	3310 (4 3 5)	3315 (5 2 0)
Birth length, cm	50.7 (2.5)	49.5 (3.4)	51.1 (3.5)	49.9 (3.0)	49.9 (3.9)	50.7 (3.3)
Head circumference, cm	33.0 (1.6)	33.6 (1.7)	33.8 (1.7)	33.2 (1.6)	33.5 (1.8)	33.5 (1.6)

Continuous variables are expressed as mean with standard deviation. Characteristics for the original study population have been reported previously [3].

Nineteen percent (1 7 4) of 904 saliva samples available for lactose assay were contaminated with breast milk (lactose-positive) and excluded from IgA analyses. The proportion of contaminated samples declined with age (Supplementary Table 1). Salivary total protein levels tended to decline between 1 and 4 months of age, followed by an increase (Supplementary Fig. 1). Age-related changes in total protein levels were comparable for all three study groups.

**Fig. 1:**
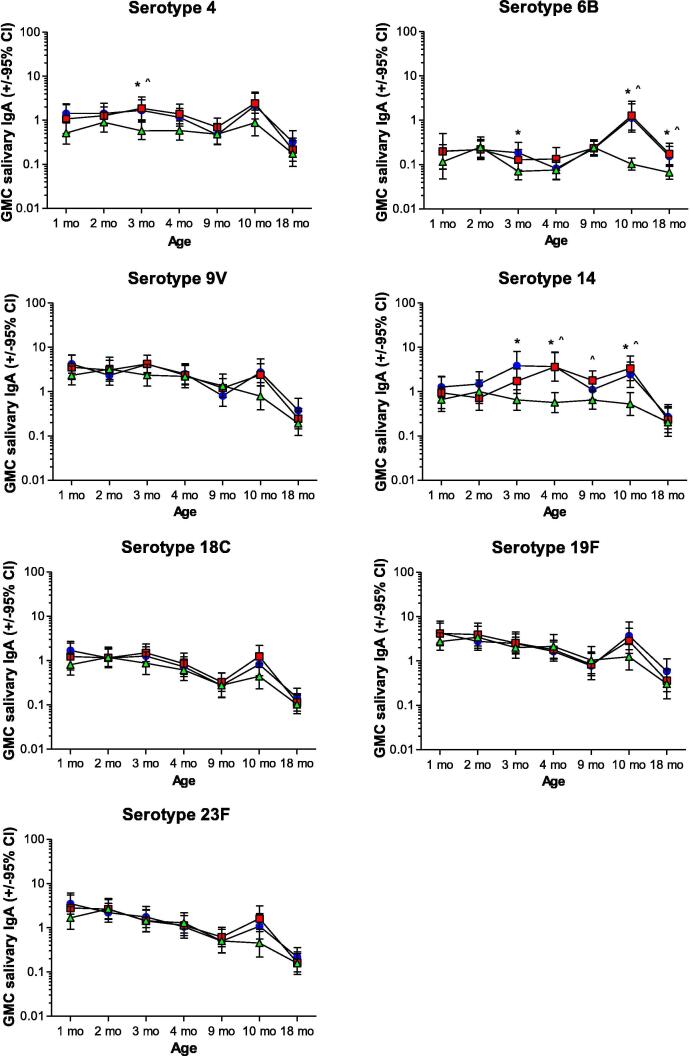
**Geometric mean concentrations of 7vPCV serotype-specific salivary IgA responses.** Shown are geometric mean concentrations (GMCs; µg antibody/mg total protein) and 95% confidence intervals for IgA antibodies against 7vPCV serotypes measured at ages 1, 2, 3, 4, 9, 10 and 18 months for the 7vPCV-primed (neonatal and infant groups) and unprimed (control) groups. Blue circles = Neonatal 7vPCV group; Red squares = Infant 7vPCV group; Green triangles = Control group. Significant differences (p-value ≤ 0.05) between the neonatal 7vPCV and control group are indicated by (*) and between the infant 7vPCV and control group with (^). (For interpretation of the references to colour in this figure legend, the reader is referred to the web version of this article.)

### 3.2 Salivary IgA responses in the first 9 months of life in relation to 7vPCV vaccination

At 1 month of age proportions of children with detectable serotype-specific IgA antibodies were comparable for the three groups (Supplementary Table 2). Detection rates in the total study population were >75% for all serotypes tested, except for serotype 6B (55% detection) (Supplementary Table 2). Detection rates for 7vPCV and non-7vPCV serotype-specific IgA declined during the first 9 months of life, both in children who did or did not receive 7vPCV.

**Table 2:** Salivary pneumococcal serotype-specific IgA and IgG geometric mean concentrations (GMC) by age in the two 7vPCV vaccine groups and controls.

	Age	IgA (GMC, and 95% CI)			IgG (GMC, and 95% CI)		
		Neonatal	Infant	Control	Neonatal	Infant	Control
**7vPCV serotypes**							
**4**	1 m	1.46 (0.90–2.36)	1.07 (0.50–2.29)	0.51 (0.29–0.92)	0.18 (0.11–0.28)	0.11 (0.07–0.19)	0.10 (0.06–0.18)
	2 m	1.43 (0.86–2.37)	1.27 (0.80–2.02)	0.90 (0.54–1.51)	0.14 (0.09–0.22)	0.11 (0.08–0.14)	0.11 (0.08–0.15)
	3 m	**1.68 (0.96**–**2.94)**	**1.87 (1.03**–**3.41)**	**0.58 (0.37**–**0.91)**	**0.29 (0.18**–**0.46)**	**0.32 (0.19**–**0.51)**	**0.11 (0.08**–**0.15)**
	4 m	1.27 (0.74–1.85)	1.40 (0.83–2.34)	0.59 (0.36–0.97)	**0.24 (0.16**–**0.36)**	***0.47 (0.29***–***0.75)***	***0.08 (0.07***–***0.11)***
	9 m	0.47 (0.29–0.77)	0.70 (0.44–1.13)	0.48 (0.28–0.83)	**0.23 (0.15**–**0.35)**	***0.40 (0.26***–***0.61)***	***0.09 (0.06***–***0.12)***
	10 m	2.17 (1.08–4.36)	2.42 (1.44–4.08)	0.88 (0.45–1.72)	**3.10 (1.83**–**5.24)**	**2.83 (1.67**–**4.80)**	**0.11 (0.07**–**0.18)**
	18 m	0.32 (0.18–0.58)	0.22 (0.12–0.40)	0.18 (0.09–0.34)	**0.29 (0.18**–**0.46)**	**0.27 (0.18**–**0.40)**	**0.11 (0.07**–**0.16)**
**6B**	1 m	0.20 (0.08–0.51)	0.20 (0.08–0.50)	0.12 (0.05–0.28)	0.39 (0.25–0.62)	0.19 (0.14–0.26)	0.20 (0.14–0.29)
	2 m	0.22 (0.14–0.34)	0.22 (0.13–0.37)	0.25 (0.15–0.42)	*0.21 (0.15*–*0.29)*	0.17 (0.14–0.20)	0.20 (0.16–0.26)
	3 m	**0.19 (0.11**–**0.32**)	0.13 (0.08–0.22)	**0.07 (0.05**–**0.11)**	*0.16 (0.13*–*0.21)*	0.16 (0.13–0.19)	0.19 (0.15–0.25)
	4 m	0.08 (0.05–0.15)	0.13 (0.07–0.24)	0.08 (0.05–0.13)	*0.25 (0.20*–*0.31)*	0.24 (0.20–0.29)	0.22 (0.19–0.24)
	9 m	0.23 (0.16–0.34)	0.23 (0.16–0.34)	0.25 (0.17–0.36)	***0.19 (0.14***–***0.25)***	**0.20 (0.14**–**0.28)**	**0.13 (0.11**–**0.14)**
	10 m	**1.12 (0.54**–**2.34)**	**1.27 (0.60**–**2.68)**	**0.10 (0.08**–**0.14)**	**1.89 (1.05**–**3.41)**	**2.90 (1.70**–**4.93)**	**0.37 (0.34**–**0.40)**
	18 m	**0.15 (0.09**–**0.26)**	**0.17 (0.10**–**0.30)**	**0.07 (0.05**–**0.09)**	**0.43 (0.29**–**0.65)**	**0.42 (0.31**–**0.59)**	**0.21 (0.19**–**0.24)**
**9V**	1 m	4.22 (2.63–6.76)	3.57 (1.90–6.69)	2.33 (1.42–3.82)	1.80 (1.19–2.72)	1.56 (1.03–2.38)	1.47 (0.94–2.30)
	2 m	2.34 (1.39–3.92)	3.08 (1.88–5.05)	3.22 (1.71–6.05)	0.85 (0.52–1.40)	0.89 (0.59–1.34)	*1.05 (0.70*–*1.56)*
	3 m	4.11 (2.55–6.63)	4.22 (2.68–6.63)	2.35 (1.33–4.13)	1.64 (1.11–2.44)	1.11 (0.77–1.60)	*0.71 (0.43*–*1.17)*
	4 m	2.53 (1.50–4.27)	2.35 (1.41–3.90)	2.21 (1.22–4.00)	*1.20 (0.78*–*1.82)*	1.25 (0.82–1.92)	*0.72 (0.48*–*1.08)*
	9 m	*0.80 (0.47*–*1.38)*	1.21 (0.74–1.96)	1.36 (0.75–2.48)	*0.88 (0.58*–*1.34)*	1.07 (0.75–1.53)	*0.47 (0.29*–*0.78)*
	10 m	2.74 (1.37–5.47)	2.38 (1.34–4.24)	0.79 (0.39–1.60)	**5.53 (3.54**–**8.65)**	**3.30 (2.09**–**5.23)**	**0.54 (0.35**–**0.85)**
	18 m	0.38 (0.20–0.71)	0.25 (0.14–0.42)	0.19 (0.10–0.36)	0.73 (0.48–1.11)	0.54 (0.35–0.83)	0.30 (0.17–0.53)
**14**	1 m	1.28 (0.73–2.22)	0.96 (0.42–2.16)	0.67 (0.36–1.24)	0.67 (0.42–1.07)	0.89 (0.59–1.32)	0.69 (0.42–1.15)
	2 m	1.48 (0.78–2.81)	0.72 (0.38–1.37)	1.00 (0.54–1.84)	*0.25 (0.17*–*0.37)*	*0.28 (0.19*–*0.42)*	*0.29 (0.19*–*0.46)*
	3 m	**3.86 (1.87**–**8.01)**	1.72 (0.92–3.25)	**0.65 (0.38**–**1.11)**	*0.55 (0.38*–*0.79)*	*0.42 (0.34*–*0.51)*	*0.45 (0.35*–*0.59)*
	4 m	***3.67 (1.72***–***7.87)***	***3.61 (1.73***–***7.54)***	**0.57 (0.34**–**0.96)**	**0.65 (0.45**–**0.95)**	*0.41 (0.34*–*0.50)*	***0.36 (0.32***–***0.41)***
	9 m	1.11 (0.67–1.85)	**1.80 (1.09**–**2.97)**	**0.64 (0.41**–**1.03)**	**0.52 (0.35**–**0.78)**	**0.59 (0.40**–**0.88)**	***0.28 (0.21***–***0.36)***
	10 m	**2.42 (1.25**–**4.69)**	**3.36 (1.78**–**6.31)**	**0.53 (0.29**–**0.95)**	**1.35 (0.82**–**2.23)**	**1.60 (1.02**–**2.52)**	**0.40 (0.32**–**0.51)**
	18 m	0.27 (0.14–0.52)	0.23 (0.12–0.46)	0.20 (0.10–0.43)	0.30 (0.21–0.44)	0.27 (0.20–0.37)	0.22 (0.16–0.30)
**18C**	1 m	1.70 (1.07–2.69)	1.24 (0.62–2.47)	0.81 (0.47–1.39)	0.61 (0.41–0.89)	0.60 (0.44–0.83)	0.47 (0.30–0.72)
	2 m	1.15 (0.69–1.91)	1.16 (0.73–1.82)	1.18 (0.69–2.03)	0.36 (0.23–0.58)	*0.22 (0.15*–*0.34)*	*0.24 (0.15*–*0.38)*
	3 m	1.26 (0.78–2.03)	1.49 (0.94–2.36)	0.88 (0.49–1.57)	**0.47 (0.28**–**0.77)**	**0.44 (0.29**–**0.65)**	***0.20 (0.12***–***0.31)***
	4 m	0.73 (0.44–1.21)	0.85 (0.49–1.47)	0.62 (0.35–1.07)	**0.45 (0.28**–**0.71)**	***0.86 (0.54***–***1.37)***	***0.14 (0.10***–***0.21)***
	9 m	0.28 (0.14–0.52)	0.33 (0.20–0.53)	*0.28 (0.15*–*0.52*)	0.33 (0.20–0.54)	**0.54 (0.37**–**0.79)**	***0.19 (0.14***–***0.27)***
	10 m	0.82 (0.46–1.47)	1.24 (0.71–2.19)	0.44 (0.23–0.85)	**2.20 (1.23**–**3.97)**	**2.21 (1.23**–**3.95)**	**0.21 (0.13**–**0.34)**
	18 m	0.15 (0.09–0.24)	0.11 (0.07–0.18)	0.10 (0.06–0.17)	**0.25 (0.15**–**0.42)**	**0.27 (0.17**–**0.43)**	**0.10 (0.06**–**0.15)**
**19F**	1 m	4.27 (2.57–7.10)	4.20 (2.20–7.99)	2.75 (1.74–4.33)	1.24 (0.77–1.98)	0.80 (0.48–1.32)	0.84 (0.49–1.46)
	2 m	2.76 (1.75–4.37)	3.94 (2.18–7.11)	3.44 (1.95–6.08)	*0.74 (0.51*–*1.09)*	0.52 (0.35–0.78)	*0.49 (0.31*–*0.76)*
	3 m	2.48 (1.52–4.05)	2.55 (1.45–4.49)	2.00 (1.16–3.46)	0.99 (0.64–1.52)	0.74 (0.52–1.06)	*0.51 (0.34*–*0.76)*
	4 m	1.64 (0.99–2.71)	1.80 (1.10–2.95)	2.13 (1.15–3.94)	0.68 (0.43–1.08)	0.74 (0.50–1.09)	*0.39 (0.27*–*0.55)*
	9 m	0.78 (0.38–1.60)	*0.83 (0.47*–*1.49)*	1.05 (0.52–2.11)	*0.36 (0.22*–*0.58)*	*0.51 (0.31*–*0.85)*	0.29 (0.18–0.46)
	10 m	3.69 (1.80–7.55)	2.82 (1.47–5.41)	1.25 (0.63–2.46)	**2.31 (1.14**–**4.67)**	**2.14 (1.08**–**4.25)**	**0.29 (0.19**–**0.44)**
	18 m	0.59 (0.31–1.12)	0.36 (0.20–0.64)	0.31 (0.14–0.66)	0.48 (0.27–0.84)	0.36 (0.22–0.58)	0.22 (0.14–0.35)
**23F**	1 m	3.52 (2.02–6.11)	2.82 (1.44–5.50)	1.68 (0.93–3.04)	0.87 (0.60–1.27)	0.57 (0.41–0.80)	0.59 (0.39–0.90)
	2 m	2.19 (1.34–3.57)	2.64 (1.60–4.36)	2.66 (1.55–4.58)	*0.42 (0.27*–*0.66)*	*0.29 (0.21*–*0.41)*	*0.37 (0.24*–*0.55)*
	3 m	1.75 (1.01–3.04)	1.43 (0.80–2.56)	1.44 (0.82–2.50)	*0.40 (0.26*–*0.61)*	*0.28 (0.21*–*0.38)*	*0.29 (0.19*–*0.43)*
	4 m	*1.05 (0.58*–*1.89)*	*1.12 (0.67*–*1.89)*	1.29 (0.76–2.18)	***0.34 (0.22***–***0.51)***	**0.41 (0.26**–**0.63)**	***0.18 (0.13***–***0.26)***
	9 m	*0.50 (0.27*–*0.93)*	*0.62 (0.37*–*1.02)*	*0.50 (0.28*–*0.93)*	*0.29 (0.18*–*0.46)*	*0.30 (0.20*–*0.45)*	0.17 (0.12–0.25)
	10 m	1.08 (0.56–2.09)	1.61 (0.82–3.15)	0.45 (0.22–0.93)	**0.95 (0.51**–**1.76)**	**2.10 (1.11**–**3.96)**	**0.13 (0.09**–**0.18)**
	18 m	0.22 (0.13–0.36)	0.16 (0.10–0.26)	0.16 (0.09–0.29)	0.26 (0.16–0.41)	0.26 (0.17–0.38)	0.14 (0.09–0.21)
**Non-7vPCV serotypes**							
**1**	1 m	3.16 (2.08–4.82)	2.44 (1.32–4.52)	2.03 (1.34–3.07)	1.06 (0.68–1.65)	0.69 (0.44–1.07)	0.71 (0.43–1.17)
	2 m	1.51 (0.91–2.50)	2.09 (1.39–3.37)	2.20 (1.26–3.84)	*0.49 (0.31*–*0.78)*	0.52 (0.35–0.78)	0.40 (0.25–0.65)
	3 m	1.43 (0.88–2.33)	1.36 (0.80–2.30)	1.35 (0.78–2.33)	*0.35 (0.23*–*0.53)*	*0.32 (0.24*–*0.44)*	*0.35 (0.22*–*0.57)*
	4 m	1.18 (0.77–1.83)	1.03 (0.63–1.69)	1.18 (0.68–2.05)	*0.29 (0.18*–*0.48)*	*0.19 (0.13*–*0.28)*	*0.27 (0.17*–*0.43)*
	9 m	*0.44 (0.25*–*0.76)*	*0.64 (0.40*–*1.03)*	0.68 (0.36–1.30)	*0.20 (0.12*–*0.32)*	*0.20 (0.13*–*0.29)*	*0.29 (0.18*–*0.46)*
	10 m	0.96 (0.48–1.94)	0.97 (0.53–1.78)	0.78 (0.37–1.63)	0.19 (0.13–0.29)	0.19 (0.13–0.29)	0.30 (0.18–0.51)
	18 m	0.19 (0.11–0.34)	0.16 (0.10–0.28)	0.16 (0.08–0.31)	0.15 (0.09–0.26)	0.11 (0.07–0.17)	0.13 (0.07–0.23)
**5**	1 m	1.38 (0.76–2.51)	1.10 (0.53–2.28)	0.71 (0.38–1.33)	0.54 (0.35–0.82)	0.40 (0.27–0.60)	0.39 (0.24–0.65)
	2 m	0.75 (0.43–1.33)	0.82 (0.46–1.50)	1.22 (0.67–2.44)	*0.28 (0.19*–*0.42)*	0.23 (0.16–0.34)	0.24 (0.15–0.38)
	3 m	0.81 (0.53–1.25)	0.75 (0.49–1.14)	0.85 (0.49–1.49)	*0.18 (0.11*–*0.28)*	*0.15 (0.11*–*0.22)*	*0.19 (0.12*–*0.32)*
	4 m	0.72 (0.40–1.27)	0.52 (0.29–0.93)	0.71 (0.37–1.35)	*0.17 (0.11*–*0.26)*	*0.11 (0.08*–*0.16)*	*0.18 (0.11*–*0.28)*
	9 m	0.29 (0.16–0.54)	0.42 (0.25–0.71)	0.32 (0.17–0.61)	*0.11 (0.07*–*0.17)*	*0.11 (0.07*–*0.17)*	*0.17 (0.10*–*0.27)*
	10 m	0.90 (0.47–1.71)	1.17 (0.65–2.10)	1.23 (0.63–2.38)	0.13 (0.08–0.20)	0.13 (0.08–0.21)	0.20 (0.11–0.37)
	18 m	0.18 (0.10–0.34)	0.17 (0.10–0.32)	0.13 (0.06–0.27)	0.14 (0.09–0.22)	0.12 (0.08–0.18)	0.13 (0.08–0.22)
**7F**	1 m	2.55 (1.68–3.87)	1.97 (1.03–3.79)	1.21 (0.72–2.03)	0.74 (0.49–1.13)	0.50 (0.32–0.76)	0.48 (0.28–0.83)
	2 m	1.24 (0.78–1.97)	1.67 (1.03–2.73)	1.83 (1.06–3.16)	*0.40 (0.27*–*0.61)*	0.38 (0.26–0.54)	*0.30 (0.19*–*0.48)*
	3 m	1.18 (0.72–1.94)	0.99 (0.59–1.67)	1.11 (0.63–1.95)	*0.25 (0.15*–*0.41)*	0.18 (0.12–0.26)	0.24 (0.14–0.43)
	4 m	1.03 (0.61–1.74)	0.93 (0.55–1.57)	1.13 (0.65–1.98)	*0.24 (0.15*–*0.40)*	*0.12 (0.08*–*0.18)*	*0.21 (0.13*–*0.34)*
	9 m	0.41 (0.21–0.82)	0.71 (0.43–1.18)	0.70 (0.38–1.29)	*0.18 (0.10*–*0.30)*	*0.20 (0.14*–*0.29)*	*0.20 (0.12*–*0.34)*
	10 m	1.46 (0.80–2.68)	1.76 (1.01–3.05)	2.20 (1.14–4.26)	0.23 (0.14–0.37)	0.18 (0.12–0.27)	0.25 (0.13–0.46)
	18 m	0.41 (0.25–0.67)	0.32 (0.20–0.52)	0.35 (0.20–0.61)	0.18 (0.11–0.30)	0.14 (0.10–0.21)	0.16 (0.10–0.27)
**19A**	1 m	4.24 (2.41–7.45)	2.67 (1.28–5.57)	2.20 (1.08–4.50)	0.46 (0.24–0.85)	0.40 (0.22–0.73)	0.33 (0.17–0.65)
	2 m	1.69 (0.98–2.93)	2.93 (1.55–4.81)	2.81 (1.51–5.25)	*0.32 (0.22*–*0.48)*	0.34 (0.22–0.52)	0.26 (0.18–0.38)
	3 m	1.64 (0.93–2.88)	1.89 (1.13–3.14)	1.16 (0.57–2.34)	*0.28 (0.20*–*0.40)*	0.29 (0.21–0.39)	0.33 (0.23–0.47)
	4 m	1.39 (0.88–2.18)	1.55 (0.95–2.52)	1.94 (1.16–3.25)	*0.26 (0.18*–*0.38)*	*0.20 (0.16*–*0.24)*	*0.21 (0.16*–*0.29)*
	9 m	0.66 (0.40–1.12)	0.93 (0.61–1.44)	1.23 (0.71–2.15)	*0.23 (0.17*–*0.29)*	*0.27 (0.20*–*0.35)*	0.26 (0.19–0.36)
	10 m	0.70 (0.38–1.26)	0.54 (0.33–0.90)	0.40 (0.21–0.78)	0.12 (0.10–0.14)	0.17 (0.13–0.22)	0.12 (0.10–0.15)
	18 m	0.17 (0.09–0.29)	0.13 (0.09–0.21)	0.13 (0.08–0.22)	0.14 (0.10–0.21)	0.14 (0.11–0.17)	0.17 (0.12–0.24)

Salivary antibody responses were standardized as µg per mg of total protein (µg antibody/mg total protein). Responses at 2, 3, 4, or 9 months of age were compared to those at baseline (at 1 month of age) using the Wilcoxon signed-rank test, and expressed in *italics* when found to be significantly different (p ≤ 0.05). GMCs and 95% CIs expressed in **bold** indicate significant differences in responses between 7vPCV-vaccinated children (neonatal and/or infant group) and the controls group based on the 95% CIs.

**Fig. 2:**
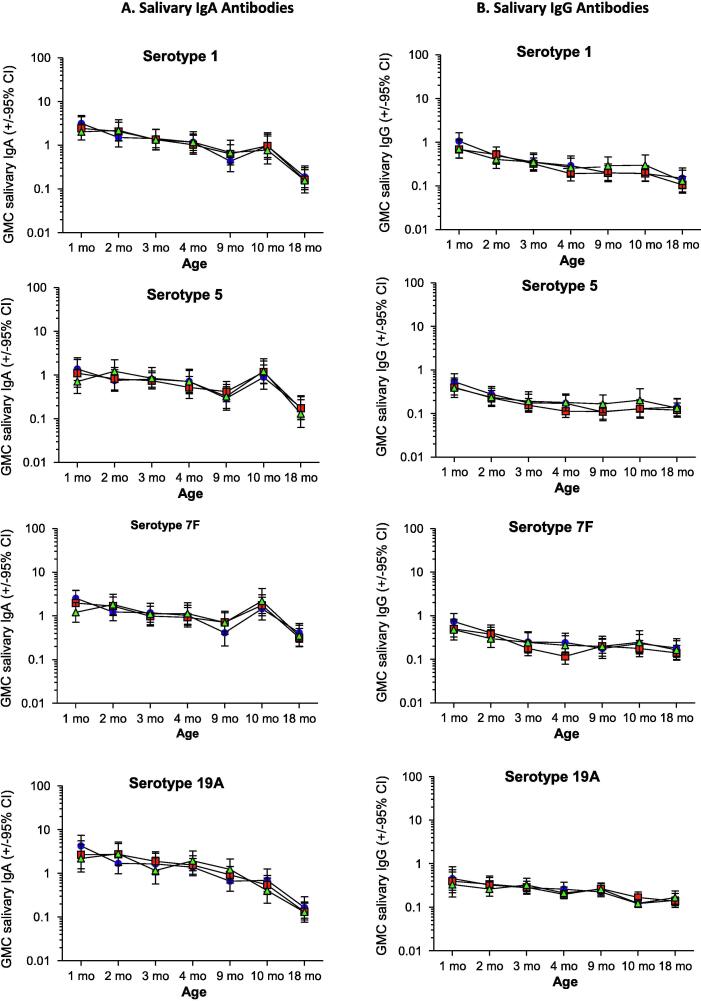
**Geometric mean concentrations salivary IgA and IgG responses against non-7vPCV serotypes.** Geometric mean concentrations (GMCs; µg antibody/mg total protein) and 95% confidence intervals for a) IgA and b) IgG antibodies against four non-7vPCV serotypes measured at ages 1, 2, 3, 4, 9, 10 and 18 months for the PCV-primed (neonatal and infant groups) and unprimed (control) groups. Blue circles = Neonatal 7vPCV group; Red squares = Infant 7vPCV group; Green triangles = Control group. (For interpretation of the references to colour in this figure legend, the reader is referred to the web version of this article.)

IgA GMCs for 7vPCV and non-7vPCV serotypes remained stable or decreased between 1 and 9 months of age in all three groups, except for an increase in serotype 14-specific IgA between 1 and 4 months of age in children vaccinated with 7vPCV (Fig. 1, Fig. 2A, Table 2). Compared to controls, GMCs of salivary IgA were significantly higher for serotype 4 at 3 months of age in the neonatal and infant 7vPCV groups; for IgA against serotype 6B at 3 months of age in the neonatal 7vPCV group; and for IgA against serotype 14 at 3 months of age in the neonatal 7vPCV group, at 4 months of age in both the neonatal and infant 7vPCV groups, and at 9 months of age in the infant 7vPCV group (Fig. 1, Fig. 2A, Table 2).

### 3.3 Salivary IgG responses in the first 9 months of life in relation to 7vPCV vaccination

At 1 month of age detection rates of serotype-specific IgG antibodies were comparable in the three groups (Supplementary Table 2). Detection rates in the total study population were >70% for all serotypes other than 6B, 14 and 19A. The proportions of children with detectable salivary IgG antibodies against 7vPCV or non-7vPCV serotypes decreased over the first 9 months of life (Supplementary Table 2). Compared to the control group, detection rates for salivary IgG remained significantly higher in both the neonatal and infant 7vPCV groups for serotype 4 at 4 and 9 months and for serotype 14 at 9 months of age, and in the infant 7vPCV group for serotype 18C at 4 and 9 months of age (Supplementary Table 2).

**Fig. 3:**
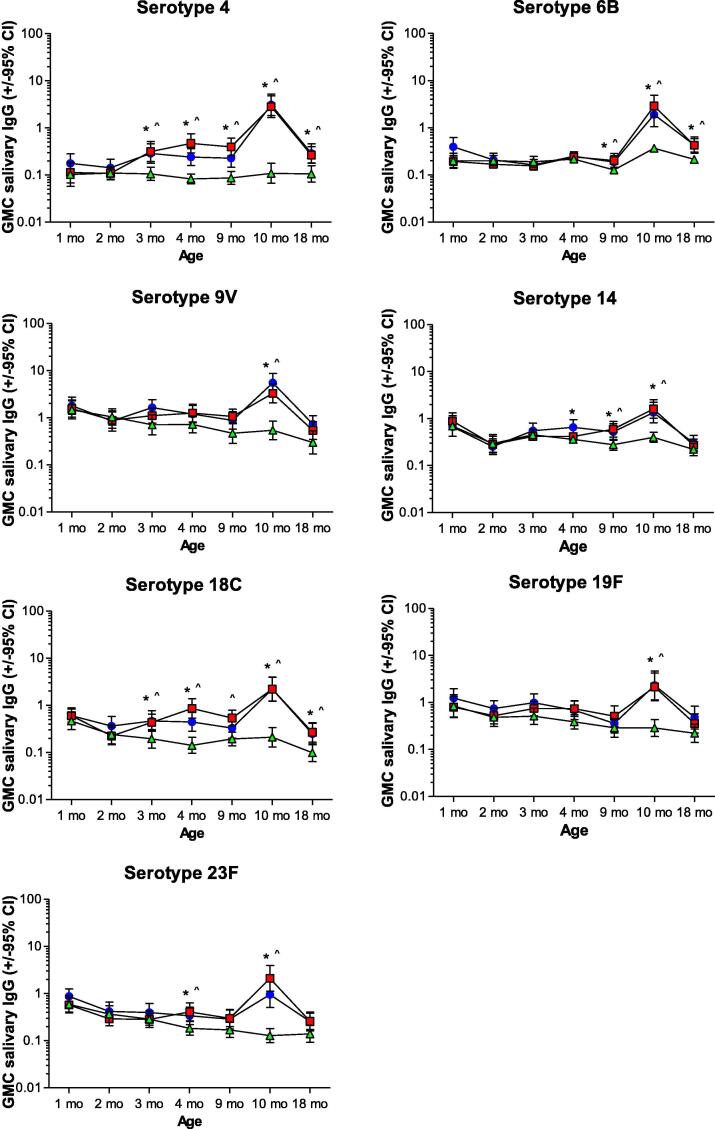
**Geometric mean concentrations of 7vPCV serotype-specific salivary IgG responses.** Geometric mean concentrations (GMCs; µg antibody/mg total protein) and 95% confidence intervals for IgG antibodies against 7vPCV serotypes measured at ages 1, 2, 3, 4, 9, 10 and 18 months for the 7vPCV-primed (neonatal and infant groups) and unprimed (control) groups. Blue circles = Neonatal 7vPCV group; Red squares = Infant 7vPCV group; Green triangles = Control group. Significant differences (p-value ≤ 0.05) between the neonatal 7vPCV and control group are indicated by (*) and between the infant 7vPCV and control group with (^). (For interpretation of the references to colour in this figure legend, the reader is referred to the web version of this article.)

In the control group salivary IgG GMCs for 7vPCV and non-7vPCV serotypes decreased between 1 and 9 months of age (Fig. 2B and 3, Table 2). In 7vPCV recipients, salivary IgG GMCs for 7vPCV serotypes increased or remained stable after 2 months of age. Consequently 7vPCV-vaccinated children had significantly higher GMCs of salivary IgG than the control group for: serotype 4 at 3, 4 and 9 months of age; for serotype 6B at 9 months of age; for serotype 14 at 4 months of age in the neonatal group, and at 9 months of age in both 7vPCV groups; for serotype 18C at 3 and 4 months of age, and at 9 months of age in the infant group; and for serotype 23F at 4 months of age. For serotypes not included in 7vPCV, GMCs of IgG were comparable for 7vPCV-vaccinated children and controls.

### 3.4 Salivary IgA and IgG responses after 23vPPV vaccination

Geometric mean ratios (GMRs) of salivary IgA and IgG antibodies at 10 versus 9 months of age (post- versus pre-23vPPV) are presented in Table 3. In 7vPCV-vaccinated children salivary IgA and IgG against 7vPCV serotypes increased significantly after 23vPPV vaccination, with the exception of serotype 14-specific IgA in the infant 7vPCV group. In the control group, salivary IgA and IgG against 7vPCV serotypes did not change after 23vPPV vaccination, except for an increase in IgG and decrease in IgA antibodies against serotype 6B (Table 3). For non-7vPCV serotypes included in 23vPPV, salivary IgA responses against serotypes 5 and 7F increased significantly after 23vPPV vaccination in all groups, and so did IgA antibodies against serotype 1 in the neonatal 7vPCV group (Table 3). In contrast, salivary IgA against serotype 19A decreased significantly in the infant 7vPCV and control group, and so did IgG responses in the neonatal and infant 7vPCV groups after 23vPPV vaccination.

**Table 3:** Geometric mean ratios (GMRs) of salivary IgA and IgG responses after 23vPPV vaccination in two groups of children previously vaccinated with 7vPCV and in controls.

	Salivary IgA − 10 month / 9 month			Salivary IgG − 10 month / 9 month		
	7vPCV groups			7vPCV groups		
	Neonatal	Infant	Control	Neonatal	Infant	Control
**7vPCV serotypes**						
4	4.40 (2.18–8.90)	3.31 (2.01–5.46)	1.53 (0.80–2.95)	13.58 (7.02–26.27)	8.05 (4.38–14.79)	1.69 (0.52–5.46)
6B	4.75 (1.97–11.43)	5.07 (2.61–9.82)	0.31 (0.22–0.44)	10.10 (5.75–17.74)	15.64 (9.00–27.19)	3.67 (1.39–9.70)
9V	3.86 (1.81–8.24)	2.18 (1.15–4.14)	0.57 (0.29–1.10)	6.28 (3.66–10.78)	3.88 (2.34–6.41)	1.49 (0.52–4.28)
14	2.41 (1.30–4.47)	1.68 (0.90–3.15)	0.72 (0.43–1.21)	2.58 (1.68–3.95)	3.01 (1.69–5.36)	1.86 (0.72–4.85)
18C	3.51 (1.65–7.45)	3.41 (1.87–6.19)	1.47 (0.75–2.86)	6.67 (3.53–12.60)	4.58 (2.31–9.06)	1.44 (0.49–4.30)
19F	6.12 (2.71–13.81)	2.69 (1.30–5.59)	1.21 (0.60–2.44)	6.42 (2.95–13.95)	4.61 (2.43–8.72)	1.31 (0.42–4.05)
23F	2.52 (1.09–5.82)	2.47 (1.08–5.68)	0.81 (0.33–1.98)	3.31 (1.66–6.59)	8.20 (4.21–15.94)	1.02 (0.33–3.17)
**Non-7vPCV serotypes**						
1	2.69 (1.30–5.56)	1.39 (0.76–2.54)	1.22 (0.60–2.48)	0.96 (0.60–1.53)	1.21 (0.62–2.23)	1.36 (0.44–4.17)
5	3.20 (1.60–6.40)	2.85 (1.43–5.68)	4.22 (1.93–9.20)	1.18 (0.72–1.94)	1.48 (0.76–2.90)	1.61 (0.47–5.47)
7F	3.60 (1.86–6.95)	2.51 (1.32–4.80)	3.26 (1.87–5.67)	1.29 (0.77–2.18)	1.09 (0.64–1.85)	1.62 (0.48–5.49)
19A	1.15 (0.65–2.04)	0.52 (0.32–0.85)	0.30 (0.16–0.57)	0.62 (0.49–0.79)	0.68 (0.56–0.82)	0.53 (0.16–1.76)

Presented are the geometric mean ratios (GMRs) and 95% confidence intervals of fold-changes in serotype-specific antibody levels at 10 months compared to 9 months of age. GMRs > 1 with 95% CIs not including 1 indicate significant increases in antibody responses, whereas GMRs < 1 with 95% CIs not including 1 indicate significant decreases. All children received one dose of 23vPPV at 9 months of age. All serotypes for which salivary IgA and IgG antibodies were measured are included in 23vPPV.

Comparing antibody levels one month after 23vPPV vaccination between 7vPCV recipients and controls, IgA and IgG GMCs for 7vPCV serotypes were higher in 7vPCV-vaccinated children than controls. However, for IgA this was only significant for serotypes 6B and 14 (Fig. 1, Fig. 3, Table 2).

### 3.5 Waning of salivary IgA and IgG responses

By 18 months of age salivary IgA and IgG responses to 7vPCV and non-7vPCV serotypes had decreased to levels comparable to or lower than before 23vPPV vaccination, except for serotype 6B-specific salivary IgG responses that were higher at 18 than at 9 months of age in the 7vPCV-vaccinated groups (Fig. 1, Fig. 2, Fig. 3 and Table 2). Compared to the control group, GMCs of salivary IgA against serotype 6B and IgG against 7vPCV serotypes 4, 6B and 18C remained significantly higher at 18 months of age in 7vPCV-vaccinated children (neonatal and infant groups). For non-7vPCV serotypes, GMCs for IgG and IgA in saliva were comparable for 7vPCV recipients and controls at 18 months of age.

### 3.6 Correlations between serum IgG and salivary IgG

Correlations between salivary IgG and serum IgG against all 7vPCV serotypes and non-7vPCV serotypes 5 and 7F at 4, 10 and 18 months of age were studied for 7vPCV-vaccinated children (neonatal and infant groups combined) and controls (serum IgG was assessed as part of the main trial and published previously [3]) (Table 4). At 4 months of age, all serum and salivary 7vPCV-serotype-specific IgG antibodies other than serotype 14 were significantly correlated for 7vPCV-vaccinated children; no correlations were found for non-7vPCV serotypes in 7vPCV-vaccinated children; and no correlations were found for 7vPCV or non-7PCV serotypes in the control group. One month after 23vPPV vaccination, 7vPCV-serotype-specific (but not non-7vPCV serotype-specific) serum and salivary IgG responses were strongly correlated in 7vPCV recipients, and in the control group serum and salivary IgG antibodies were correlated for 7vPCV serotypes 4 and 18C and non-7vPCV serotypes 5 and 7F. At 18 months of age, no correlations were found in the 7vPCV-vaccinated children, whereas in the controls serum and salivary IgG antibodies were negatively correlated for 7vPCV serotypes 6B and 19F.

**Table 4:** Correlation coefficients between serum and salivary IgG responses in children vaccinated with 7vPCV and in controls.

	4 months of age		10 months of age		18 months of age	
	7vPCV	Control	7vPCV	Control	7vPCV	Control
**7vPCV serotypes**						
4	0.382**	0.164	0.627**	0.334**	0.179	−0.195
6B	0.219**	0.068	0.695**	0.184	0.203	−0.332**
9V	0.321**	−0.170	0.534**	0.252	−0.017	−0.297
14	0.154	−0.012	0.591**	0.208	−0.186	−0.327
18C	0.266**	−0.088	0.664**	0.493**	0.185	−0.222
19F	0.208**	−0.124	0.605**	0.333	0.073	−0.420**
23F	0.369**	−0.069	0.807**	0.017	0.129	−0.303
**Non-7vPCV serotypes**						
5	0.087	−0.057	−0.019	0.525**	−0.041	−0.193
7F	0.144	−0.221	0.167	0.356**	−0.084	−0.252

Since salivary antibody responses were comparable for children at 4 months of age and older after completing vaccination with 3 doses of 7vPCV according to the 0–1–2 months of age (neonatal) or 1–2-3 months of age (infant) schedule, children were studied as one group (7vPCV). All children received one dose of 23vPPV at 9 months of age. Correlations were assessed using the Spearman-rank non-parametric test. Significant correlations are indicated with ** (p < 0.001).

## 4 Discussion

To our knowledge this is the first study describing mucosal antibody responses to PCV vaccination, followed by PPV booster, in children in a high-endemicity setting. This analysis of salivary antibody responses complements our earlier studies on systemic immune responses in high-risk children in PNG vaccinated according to accelerated schedules with 3 doses of PCV followed by a PPV booster at 9 months of age [3]. The study shows that immunization with 7vPCV starting as early as at birth induces mucosal IgG and IgA antibodies against some 7vPCV serotypes, and induces PCV-serotype-specific immune memory that results in production of PCV-serotype-specific salivary IgG and IgA antibodies in response to a 23vPPV booster. By the age of 3 to 4 months, accelerated 7vPCV immunization was associated with significantly higher mucosal IgA responses to three out of the seven 7vPCV serotypes (4, 6B, and 14) and mucosal IgG responses to four out of the seven 7vPCV serotypes (4, 14, 18C and 23F). Subsequent 23vPPV booster vaccination of 7vPCV-primed children resulted in enhanced salivary IgA and IgG responses against all 7vPCV serotypes, regardless of the accelerated immunization schedule, except for salivary IgA against serotype 14 for which pre-PPV levels were high and post-booster levels did not increase significantly in children vaccinated with 7vPCV at 1, 2 and 3 months of age. Finally, 23vPPV immunization induced mucosal IgA responses against two (5, 7F) out of the four 23vPPV serotypes not included in 7vPCV that were tested.

The effects of PCV on mucosal immune responses have been studied in European children at low risk for pneumococcal infections, and in general no or limited mucosal responses to PCV were found. Salivary IgA and IgG antibodies were rarely detectable in Finnish infants vaccinated with 7vPCV at 2, 4 and 6 months of age [33], while a study in the UK found elevated levels of serotype 14-specific IgA in saliva of children after vaccination with 7vPCV at 2, 3 and 5 months of age [19]. There are two possible non-exclusive explanations why, in contrast to the earlier European studies, we observe an effect of PCV on mucosal immune responses in the PNG cohort. Firstly, we measured antibody responses using a multiplex immuno-assay, which has a greater dynamic range and up to 2000 times more sensitive than the ELISA/EIA methods used in the UK and Finland studies. Secondly, compared to children in low-risk settings, PCV may be more capable of inducing mucosal immune responses in children at high risk of pneumococcal infections, even after or maybe because of our accelerated vaccination schedules. Infants in high-risk settings such as PNG experience early pneumococcal colonization with a broad range of pneumococcal serotypes [6], [7], [8]. Possibly this early colonization stimulates the development of mucosal vaccine responses, unlike systemic vaccine responses that reportedly may be suppressed in individuals colonized with vaccine serotypes before vaccination [34], [35]. Findings are more consistent for responses after booster vaccination with studies from the UK, Finland and the Netherlands reporting that PCV-serotype-specific salivary IgA and IgG responses are increased in PCV-primed children after booster vaccination with PPV or PCV [19], [21], [33]. In line with these studies in low-risk children, we have demonstrated that PCV primes for immune memory responses that result in enhanced mucosal IgA and IgG antibody responses to subsequent booster vaccination even when PCV is given at very young age.

In line with a study conducted in the Netherlands, we found that serum and saliva IgG antibodies were correlated after booster vaccination [21]. Whereas it is likely that the IgG antibodies present at moderate levels in saliva after primary PCV immunization and at higher levels after PPV booster vaccination diffused from serum, some IgG may have been produced in the upper respiratory mucosa. This is supported by the relatively greater fold increase of salivary IgG compared to serum IgG [3] antibodies after PPV booster vaccination in 7vPCV-vaccinated children. Salivary IgA antibodies are more likely to be locally secreted and, as indicated by the increase after PPV booster vaccination, are likely to be produced by PCV-induced mucosal memory cells.

Given the correlation between serum and salivary IgG antibodies, saliva could be a useful alternative sample to assess serotype-specific antibody responses after PCV vaccination. An advantage of saliva is that it is easier to collect than serum and non-invasive. Our study confirms earlier findings of Verhagen et al. who compared serum and salivary IgG responses after 13vPCV vaccination of Venezuelan Warao Amerindian children [36]. As indicated by Verhagen et al., to use saliva as a convenient sample to measure PCV-induced immunity, it is important to define cut-off levels for protection in large cohort studies and improve limits of detection and quantification of antibodies.

PCV vaccination has been shown to prevent colonization with vaccine serotypes in children in low-risk settings [17], [18], but in high-endemicity settings the impact of PCVs on carriage is limited [4], [23]. This seems to contrast with our study findings that suggest PCV is more likely to induce mucosal immune responses in children in high-risk than in low-risk settings. It has been demonstrated in human challenge models involving adult volunteers that PCV-induced salivary IgG responses and/or memory B cells can protect against pneumococcal serotype-specific acquisition [37], [38]. It is possible that in high-risk populations like the PNG infants, the density of pneumococcal colonization is too high for the PCV-induced mucosal antibody response to allow complete clearance. A limitation of our study is that we did not assess density of pneumococcal colonization and the study size is too small to properly study pneumococcal serotype-specific responses and acquisition. A larger study would be needed to better understand the impact of PCVs on mucosal immunity, pneumococcal colonization, carriage load, and herd protection in high-risk settings.

This randomized placebo-controlled vaccination trial was conducted not long after 7vPCV became available and in line with its aims successfully showed the safety and immunogenicity of this vaccine when given in accelerated schedules to high-risk children [3]. Based on these positive findings, routine childhood 13vPCV vaccination was implemented in PNG in 2014. A placebo-controlled trial as we conducted in the early 2000′s can no longer be conducted, and the results presented here are therefore unique and cannot be repeated with newer PCVs. There is, however, no reason why the effect of higher valency vaccines would be different to that of 7vPCV. We have previously shown that both PCV10 and PCV13 induce similar safety and systemic immunogenicity in PNG infants [22].

In high-risk settings like PNG, children are exposed to a very broad range of pneumococcal serotypes as indicated by the>60 different pneumococcal serotypes that we isolated from infants’ upper respiratory tract in the first year of life in two separate trials [4], [22]. This outnumbers the number of serotypes that are or could be included in PCVs. Until new PCVs including more serotypes than the current 10- and 13-valent vaccines or serotype-independent vaccines become available, 23vPPV booster vaccination could provide these high-risk children with broader protection than induced by current PCVs alone. We have previously shown that PPV induces serotype-specific systemic IgG responses in PNG infants vaccinated with PCV as well as non-PCV recipients, with no evidence of inducing hypo-responsiveness [3], [39], [40]. We here show that a PPV booster results in enhanced mucosal IgA and IgG responses in PCV-primed PNG infants, which importantly could protect children against serotype-specific acquisition and carriage. This is particularly relevant because pneumococcal carriage is almost universal in this population at least up to the age of 5 years [8]. In addition, there was evidence that PPV induced mucosal immune responses against non-PCV serotypes, but this needs more investigation to understand whether this response is related to pneumococcal serotype-specific carriage or other factors.

In summary, this study shows that in high-risk children, PCV given in early infancy (starting as early as at birth) induces mucosal antibody responses as early as at 3 to 4 months of age and importantly, primes for mucosal immune memory, as evidenced by mucosal immune responses to booster vaccination with PPV. These vaccine-induced mucosal antibody responses may reduce pneumococcal carriage, carriage density and disease.

## CRediT authorship contribution statement

**Tilda Orami:** Investigation. **Rebecca Ford:** Investigation, Formal analysis. **Lea-Ann Kirkham:** Methodology, Writing - review & editing. **Ruth Thornton:** Investigation, Methodology, Validation, Writing - review & editing. **Karli Corscadden:** Investigation, Methodology, Writing - review & editing. **Peter C. Richmond:** Conceptualization, Funding acquisition. **William S. Pomat:** Conceptualization, Funding acquisition. **Anita H.J. den Biggelaar:** Formal analysis, Writing - original draft, Writing - review & editing, Visualization. **Deborah Lehmann:** Conceptualization, Funding acquisition, Formal analysis, Data curation, Writing - review & editing, Supervision. **:** .

## Declaration of Competing Interest

The authors declare the following financial interests/personal relationships which may be considered as potential competing interests: L-A Kirkham has received educational grants and travel support from Pfizer and GSK to attend conferences, is an investigator on an investigator-initiated research grant funded by Pfizer Australia and is an inventor on patents for a pneumococcal protein vaccine antigen. P Richmond has been a member of vaccine advisory boards for Wyeth and CSL Ltd and has received institutional funding for investigator-initiated research from GlaxoSmithKline Biologicals and Merck and received travel support from Pfizer and Baxter to present study data at international meetings. D Lehmann has been a member of the GSK Australia Pneumococcal-*Haemophilus influenzae-*Protein D conjugate vaccine (“Phid-CV”) Advisory Panel, has received support from Pfizer Australia and GSK Australia to attend conferences, has received an honorarium from Merck Vaccines to give a seminar at their offices in Pennsylvania and to attend a conference, and was an investigator on an investigator-initiated research grant funded by Pfizer Australia. WS Pomat has received funding from Pfizer Australia to attend a conference. Anita H.J. van den Biggelaar has received support from Pfizer Australia and GSK Australia to attend conferences; she was previously an employee of Janssen Pharmaceuticals, Johnson & Johnson, and conducts part-time consultancy work for vaccine companies on projects not related to this study. The other authors declare to have no competing interests.
