# Supplementary material for: Pneumococcal conjugate vaccine primes mucosal immune responses to pneumococcal polysaccharide vaccine booster in Papua New Guinean children
Source: Vaccine. 2020 Nov 25;38(50):7977–88. doi: 10.1016/j.vaccine.2020.10.042 (PMC7684155; doi:10.1016/j.vaccine.2020.10.042)
Supplement: Supplementary data 1 [file mmc1.docx]

**Supplementary Table 1:** **The proportion of 904 saliva samples that were lactose-positive in the two 7vPCV groups and controls at each time point**

|  |  |  | **Age at sampling** | | | |  |  |
| --- | --- | --- | --- | --- | --- | --- | --- | --- |
| **7vPCV groups** | **1 month** | **2 months** | **3 months** | **4 months** | **9 months** | **10 months** | **18 months** | **Total** |
| **Neonatal** | 42.9% (18) | 31.0% (13) | 7.1% (3) | 23.8% (10) | 11.9% (5) | 0.0% (0) | 2.4% (1) | 17.0% (50) |
| **Infant** | 42.0% (21) | 36.0% (18) | 24.0% (12) | 15.7% (8) | 4.0% (2) | 22.9% (11) | 8.0% (4) | 21.8% (76) |
| **Control** | 33.3% (12) | 21.1% (8) | 21.1% (8) | 23.7% (9) | 15.8% (6) | 10.8% (4) | 2.8% (1) | 18.4% (48) |
| **Total** | 39.8% (51) | 30.0% (39) | 17.7% (23) | 20.6% (27) | 10.0% (13) | 11.8% (15) | 4.7% (6) | 19.2% (174) |

Proportions are expressed as % of saliva samples tested in relevant group; number of positive samples in brackets.

**Supplementary Figure 1:** **Total protein levels (and 95% confidence intervals) in saliva samples in the two 7vPCV groups and controls at different ages**

**Supplementary Table 2. Proportions of children with pneumococcal serotype-specific IgA and IgG in saliva (%) and 95% confidence intervals (95% CI) by age in the two 7vPCV vaccine groups and controls**

| **7vPCV** | **Age** | **Proportion with detectable IgA (%) (95% CI)** | | | **Proportion with detectable IgG (%) (95% CI)** | | |  |
| --- | --- | --- | --- | --- | --- | --- | --- | --- |
|  |  | **Neonatal** | **Infant** | **Control** | **Neonatal** | **Infant** | **Control** |  |
| **ST 4** | 1m | 95.8 (78.9-99.9) | 82.8 (64.2-94.2) | 78.3 (56.3-92.5) | 82.9 (67.9-92.8) | 70.6 (56.2-82.5) | 63.9 (46.2-79.2) | |
|  | 2m | 96.6 (82.2-99.9) | 93.8 (79.2-99.2) | 83.3 (65.3-94.4) | 47.4 (39.7-71.5) | 50.0 (35.5-64.5) | 47.4 (31.0-64.2) | |
|  | 3m | 89.7 (75.8-97.1) | 81.6 (65.7-92.3) | 73.3 (54.1-87.7) | 61.9 (45.6-76.4) | 66.0 (51.2-78.8) | 36.1 (20.8-53.8) | |
|  | 4m | 87.5 (71.0-96.5) | 79.1 (64.0-90.0) | 69.0 (49.2-84.7) | **64.3 (48.0-78.4)** | **76.5 (62.5-87.2)** | **28.9 (15.4-45.9)** | |
|  | 9m | 56.8 (39.5-72.9) | 66.7 (51.6-79.6) | 50.0 (31.9-68.1) | **71.4 (55.4-84.3)** | **74.0 (59.7-85.4)** | **29.7 (15.9-47.0)** | |
|  | 10m | 85.7 (71.5-94.6) | 97.3 (85.8-99.9) | 81.8 (64.5-93.0) | **95.2 (83.8-99.4)** | **96.0 (86.3-99.5)** | **44.4 (27.9-61.9)** | |
|  | 18m | 75.6 (59.7-87.6) | 67.4 (52.0-80.5) | 65.7 (47.8-80.9) | 73.2 (57.1-85.8) | 76.0 (61.8-86.9) | 45.9 (29.5-63.1) | |
| **ST 6B** | 1m | 66.7 (44.7-84.4) | 55.2 (35.7-73.6) | 43.5 (23.2-65.5) | 53.7 (37.4-69.3) | 29.4 (17.5-43.8) | 27.8 (14.2-45.2) | |
|  | 2m | 37.9 (20.7-57.7) | 31.3 (16.1-50.0) | 43.3 (25.5-62.6) | 24.4 (12.4-40.3) | 14.0 (5.8-26.7) | 18.4 (7.7-34.3) | |
|  | 3m | **46.2 (30.1-62.8)** | 34.2 (19.6-51.4) | **13.3 (3.8-30.7)** | 7.1 (1.5-19.5) | 2.0 (0.1-10.6) | 13.9 (4.7-29.5) | |
|  | 4m | 25.0 (11.5-43.4) | 34.9 (21.0-50.9) | 20.7 (8.0-39.7) | 14.3 (5.4-28.5) | 11.8 (4.4-23.9) | 5.3 (0.6-17.7) | |
|  | 9m | 32.4 (18.0-49.8) | 33.3 (20.4-48.4) | 31.3 (16.1-50.0) | 19.0 (8.6-34.1) | 18.0 (8.6-31.4) | 5.4 (0.7-18.2) | |
|  | 10m | **75.6 (59.7-87.6)** | **73.0 (55.9-86.2)** | **12.1 (3.4-28.2)** | **54.8 (38.7-70.2)** | **68.0 (53.3-80.5)** | **2.8 (0.1-14.5)** | |
|  | 18m | 36.6 (22.1-53.1) | 45.7 (30.9-61.0) | 14.3 (4.8-30.3) | **34.1 (20.1-50.6)** | **36.0 (22.9-50.8)** | **5.4 (0.7-18.2)** | |
| **ST 9V** | 1m | 100 (85.8-100) | 93.1 (77.2-99.2) | 95.7 (78.1-99.9) | 95.1 (83.5-99.4) | 90.2 (78.6-96.7) | 88.9 (73.9-96.9) | |
|  | 2m | 96.6 (82.2-99.9) | 96.9 (83.8-99.9) | 90.0 (73.5-97.9) | 82.9 (67.9-92.8) | 86.0 (73.3-94.2) | 89.5 (75.2-97.1) | |
|  | 3m | 94.9 (82.7-99.4) | 94.7 (82.3-99.4) | 86.7 (69.3-96.2) | 90.5 (77.4-97.3) | 86.0 (73.3-94.2) | 75.0 (57.8-87.9) | |
|  | 4m | 90.6 (75.0-98.0) | 86.0 (72.1-94.7) | 89.7 (72.6-97.8) | 88.1 (74.4-96.0) | 84.3 (71.4-93.0) | 81.6 (65.7-92.3) | |
|  | 9m | 62.2 (44.8-77.5) | 75.0 (60.4-86.4) | 78.1 (60.0-90.7) | 78.6 (63.2-89.7) | 84.0 (70.9-92.8) | 56.8 (39.5-72.9) | |
|  | 10m | 88.1 (74.4-96.0) | 91.9 (78.1-98.3) | 72.7 (54.5-86.7) | **97.6 (87.4-99.9)** | **94.0 (83.5-98.7)** | **72.2 (54.8-85.8)** | |
|  | 18m | 70.7 (54.5-83.9) | 63.0 (47.5-76.8) | 57.1 (39.4-73.7) | 87.8 (73.8-95.9) | 82.0 (68.6-91.4) | 64.9 (47.5-79.8) | |
| **ST 14** | 1m | 91.7 (73.0-99.0) | 69.0 (49.2-84.7) | 78.3 (56.3-92.5) | 56.1 (39.7-71.5) | 66.7 (52.1-79.2) | 52.8 (35.5-69.6) | |
|  | 2m | 86.2 (68.3-96.1) | 68.8 (50.0-83.9) | 80.0 (61.4-92.3) | 24.4 (12.4-40.3) | 28.0 (16.2-42.5) | 31.6 (17.5-48.7) | |
|  | 3m | 87.2 (72.6-95.7) | 78.9 (62.7-90.4) | 63.3 (43.9-80.1) | 26.2 (13.9-42.0) | 20.0 (10.0-33.7) | 22.2 (10.1-39.2) | |
|  | 4m | 84.4 (67.2-94.7) | 74.4 (58.8-86.5) | 58.6 (38.9-76.5) | 28.6 (15.7-44.6) | 13.7 (5.7-26.3) | 7.9 (1.7-21.4) | |
|  | 9m | 62.2 (44.8-77.5) | 79.2 (65.0-89.5) | 50.0 (31.9-68.1) | **40.5 (25.6-56.7)** | **42.0 (28.2-56.8)** | **10.8 (3.0-25.4)** | |
|  | 10m | **78.6 (63.2-89.7)** | **83.8 (68.0-93.8)** | **42.4 (25.5-60.8)** | **52.4 (36.4-68.0)** | **60.0 (45.2-73.6)** | **5.6 (0.7-18.7)** | |
|  | 18m | 61.0 (44.5-75.8) | 52.2 (36.9-67.1) | 48.6 (31.4-66.0) | 24.4 (12.4-40.3) | 22.0 (11.5-36.0) | 13.5 (4.5-28.8) | |
| **ST 18C** | 1m | 100 (85.8-100) | 86.2 (68.3-96.1) | 87.0 (66.4-97.2) | 90.2 (76.9-97.3) | 90.2 (78.6-96.7) | 75.0 (57.8-87.9) | |
|  | 2m | 96.6 (82.2-99.9) | 93.8 (79.2-99.2) | 90.0 (73.5-97.9) | 82.9 (67.9-92.8) | 68.0 (53.3-80.5) | 65.8 (48.6-80.4) | |
|  | 3m | 89.7 (75.8-97.1) | 89.5 (75.2-97.1) | 86.7 (69.3-96.2) | 71.4 (55.4-84.3) | 80.0 (66.3-90.0) | 52.8 (35.5-69.6) | |
|  | 4m | 87.5 (71.0-96.5) | 76.7 (61.4-88.2) | 79.3 (60.3-92.0) | 76.2 (60.5-87.9) | **86.3 (73.7-94.3)** | **47.4 (31.0-64.2)** | |
|  | 9m | 56.8 (39.5-72.9) | 66.7 (51.6-79.6) | 65.6 (46.8-81.4) | 64.3 (48.0-78.4) | **80.0 (66.3-90.0)** | **45.9 (29.5-63.1)** | |
|  | 10m | 81.0 (65.9-91.4) | 86.5 (71.2-95.5) | 57.6 (39.2-74.5) | **90.5 (77.4-97.3)** | **84.0 (70.9-92.8)** | **47.2 (30.4-64.5)** | |
|  | 18m | 51.2 (35.1-67.1) | 43.5 (28.9-58.9) | 34.3 (19.1-52.2) | 68.3 (51.9-81.9) | 70.0 (55.4-82.1) | 43.2 (27.1-60.5) | |
| **ST 19F** | 1m | 95.8 (78.9-99.9) | 89.7 (72.6-97.8) | 91.3 (72.0-98.9) | 87.8 (73.8-95.9) | 78.4 (64.7-88.7) | 77.8 (60.8-89.9) | |
|  | 2m | 96.6 (82.2-99.9) | 96.9 (83.8-99.9) | 90.0 (73.5-97.9) | 80.5 (65.1-91.2) | 62.0 (47.2-75.3) | 52.6 (35.8-69.0) | |
|  | 3m | 89.7 (75.8-97.1) | 86.8 (71.9-95.6) | 86.7 (69.3-96.2) | 66.7 (50.5-80.4) | 64.0 (49.2-77.1) | 47.2 (30.4-64.5) | |
|  | 4m | 87.5 (71.0-96.5) | 79.1 (64.0-90.0) | 82.8 (64.2-94.2) | 54.8 (38.7-70.2) | 60.8 (46.1-74.2) | 50.0 (33.4-66.6) | |
|  | 9m | 67.6 (50.2-82.0) | 75.0 (60.4-86.4) | 75.0 (56.6-88.5) | 54.8 (38.7-70.2) | 54.0 (39.3-68.2) | 37.8 (22.5-55.2) | |
|  | 10m | 87.8 (73.8-95.9) | 86.5 (71.2-95.5) | 75.8 (57.7-88.9) | **78.6 (63.2-89.7)** | 70.0 (55.4-82.1**)** | **41.7 (25.5-59.2)** | |
|  | 18m | 73.2 (57.1-85.8) | 69.6 (54.2-82.3) | 57.1 (39.4-73.7) | 56.1 (39.7-71.5) | 52.0 (37.4-66.3) | 40.5 (24.8-57.9) | |
| **ST 23F** | 1m | 95.8 (78.9-99.9) | 89.7 (72.6-97.8) | 87.0 (66.4-97.2) | 95.1 (83.5-99.4) | 82.4 (69.1-91.6) | 80.6 (64.0-91.8) | |
|  | 2m | 96.6 (82.2-99.9) | 93.8 (79.2-99.2) | 90.0 (73.5-97.9) | 75.6 (59.7-87.6) | 66.0 (51.2-78.8) | 68.4 (51.3-82.5) | |
|  | 3m | 87.2 (72.6-95.7) | 84.2 (68.7-94.0) | 86.7 (69.3-96.2) | 59.5 (43.3-74.4) | 64.0 (49.2-77.1) | 54.1 (36.9-70.5) | |
|  | 4m | 81.3 (63.6-92.8) | 74.4 (58.8-86.5) | 82.8 (64.2-94.2) | 59.5 (43.3-74.4) | 58.8 (44.2-72.4) | 39.5 (24.0-56.6) | |
|  | 9m | 54.1 (36.9-70.5) | 64.6 (49.5-77.8) | 59.4 (40.6-76.3) | 54.8 (38.7-70.2) | 56.0 (41.3-70.0) | 35.1 (20.2-52.5) | |
|  | 10m | 76.2 (60.5-87.9) | 81.1 (64.8-92.0) | 57.6 (39.2-74.5) | **73.8 (58.0-86.1)** | **84.0 (70.9-92.8)** | **33.3 (18.6-51.0)** | |
|  | 18m | 51.2 (35.1-67.1) | 39.1 (25.1-54.6) | 34.3 (19.1-52.2) | 63.4 (46.9-77.9) | 64.0 (49.2-77.1) | 40.5 (24.8-57.9) | |
| **Non-7vPCV** | |  |  |  |  |  |  | |
| **ST 1** | 1m | 100 (85.8-100) | 89.7 (72.6-97.8) | 95.7 (78.1-99.9) | 92.7 (80.1-98.5) | 84.3 (71.4-93.0) | 86.1 (70.5-95.3) | |
|  | 2m | 93.1 (77.2-99.2) | 96.9 (83.8-99.9) | 90.0 (73.5-97.9) | 80.5 (65.1-91.2) | 82.0 (68.6-91.4) | 71.1 (54.1-84.6) | |
|  | 3m | 87.2 (72.6-95.7) | 81.6 (65.7-92.3) | 86.7 (69.3-96.2) | 64.3 (48.0-78.4) | 72.0 (57.5-83.8) | 66.7 (49.0-81.4) | |
|  | 4m | 90.6 (75.0-98.0) | 72.1 (56.3-84.7) | 79.3 (60.3-92.0) | 64.3 (48.0-78.4) | 56.9 (42.2-70.7) | 65.8 (48.6-80.4) | |
|  | 9m | 54.1 (36.9-70.5) | 68.8 (53.7-81.3) | 65.6 (46.8-81.4) | 59.5 (43.3-74.4) | 58.0 (43.2-71.8) | 62.2 (44.8-77.5) | |
|  | 10m | 76.2 (60.5-87.9) | 83.8 (68.0-93.8) | 72.7 (54.5-86.7) | 61.9 (45.6-76.4) | 64.0 (49.2-77.1) | 66.7 (49.0-81.4) | |
|  | 18m | 56.1 (39.7-71.5) | 54.3 (39.0-69.1) | 48.6 (31.4-66.0) | 58.5 (42.1-73.7) | 56.0 (41.3-70.0) | 54.1 (36.9-70.5) | |
| **ST 5** | 1m | 91.7 (73.0-99.0) | 82.8 (64.2-94.2) | 78.3 (56.3-92.5) | 85.4 (70.8-94.4) | 78.4 (64.7-88.7) | 75.0 (57.8-87.9) | |
|  | 2m | 86.2 (68.3-96.1) | 81.3 (63.6-92.8) | 86.7 (69.3-96.2) | 70.7 (54.5-83.9) | 62.0 (47.2-75.3) | 57.9 (40.8-73.7) | |
|  | 3m | 76.9 (60.7-88.9) | 76.3 (59.8-88.6) | 76.7 (57.7-90.1) | 50.0 (34.2-65.8) | 54.0 (39.3-68.2) | 55.6 (38.1-72.1) | |
|  | 4m | 78.1 (60.0-90.7) | 62.8 (46.7-77.0) | 72.4 (52.8-87.3) | 52.4 (36.4-68.0) | 41.2 (27.6-55.8) | 52.6 (35.8-69.0) | |
|  | 9m | 56.8 (39.5-72.9) | 68.8 (53.7-81.3) | 62.5 (43.7-78.9) | 47.6 (32.0-63.6) | 48.0 (33.7-62.6) | 54.1 (36.9-70.5) | |
|  | 10m | 76.2 (60.5-87.9) | 83.8 (68.0-93.8) | 81.8 (64.5-93.0) | 59.5 (43.3-74.4) | 52.0 (37.4-66.3) | 63.9 (46.2-79.2) | |
|  | 18m | 65.9 (49.4-79.9) | 65.2 (49.8-78.6) | 54.3 (36.6-71.2) | 48.8 (32.9-64.9) | 48.0 (33.7-62.6) | 40.5 (24.8-57.9) | |
| **ST 7F** | 1m | 100 (85.8-100) | 89.7 (72.6-97.8) | 87.0 (66.4-97.2) | 92.7 (80.1-98.5) | 84.3 (71.4-93.0) | 80.6 (64.0-91.8) | |
|  | 2m | 96.6 (82.2-99.9) | 96.9 (83.8-99.9) | 90.0 (73.5-97.9) | 75.6 (59.7-87.6) | 76.0 (61.8-86.9) | 60.5 (43.4-76.0) | |
|  | 3m | 89.7 (75.8-97.1) | 84.2 (68.7-94.0) | 86.7 (69.3-96.2) | 61.9 (45.6-76.4) | 60.0 (45.2-73.6) | 63.9 (46.2-79.2) | |
|  | 4m | 90.6 (75.0-98.0) | 81.4 (66.6-91.6) | 86.2 (68.3-96.1) | 66.7 (50.5-80.4) | 43.1 (29.3-57.8) | 63.2 (46.0-78.2) | |
|  | 9m | 62.2 (44.8-77.5) | 83.3 (69.8-92.5) | 81.3 (63.6-92.8) | 54.8 (38.7-70.2) | 62.0 (47.2-75.3) | 48.6 (31.9-65.6) | |
|  | 10m | 90.5 (77.4-97.3) | 89.2 (74.6-97.0) | 90.9 (75.7-98.1) | 69.0 (52.9-82.4) | 60.0 (45.2-73.6) | 55.6 (38.1-72.1) | |
|  | 18m | 78.0 (62.4-89.4) | 71.7 (56.5-84.0) | 74.3 (56.7-87.5) | 56.1 (39.7-71.5) | 56.0 (41.3-70.0) | 54.1 (36.9-70.5) | |
| **ST 19A** | 1m | 95.8 (78.9-99.9) | 79.3 (60.3-92.0) | 82.6 (61.2-95.0) | 58.1 (39.1-75.5) | 55.8 (39.9-70.9) | 53.1 (34.7-70.9) | |
|  | 2m | 86.2 (68.3-96.1) | 84.4 (67.2-94.7) | 83.3 (65.3-94.4) | 48.4 (30.2-66.9) | 40.5 (25.6-56.7) | 30.3 (15.6-48.7) | |
|  | 3m | 74.4 (57.9-87.0) | 78.9 (62.7-90.4) | 60.0 (40.6-77.3) | 24.2 (11.1-42.3) | 23.8 (12.1-39.5) | 31.3 (16.1-50.0) | |
|  | 4m | 59.4 (40.6-76.3) | 60.5 (44.4-75.0) | 72.4 (52.8-87.3) | 24.2 (11.1-42.3) | 16.3 (6.8-30.7) | 17.6 (6.8-34.5) | |
|  | 9m | 45.9 (29.5-63.1) | 56.3 (41.2-70.5) | 64.5 (45.4-80.8) | 12.5 (3.5-29.0) | 21.4 (10.3-36.8) | 18.8 (7.2-36.4) | |
|  | 10m | 52.4 (36.4-68.0) | 48.6 (31.9-65.6) | 37.5 (21.1-56.3) | 6.3 (0.8-20.8) | 18.6 (8.4-33.4) | 6.3 (0.8-20.8) | |
|  | 18m | 29.3 (16.1-45.5) | 30.4 (17.7-45.8) | 22.9 (10.4-40.1) | 12.9 (3.6-29.8) | 11.6 (3.9-25.1) | 15.2 (5.1-31.9) | |

Proportions in **bold** indicate significant differences in responses between 7vPCV-vaccinated children (neonatal and/or infant group) versus non-7vPCV recipients (controls) based on 95% confidence intervals.
